# Multiplexed Sequence Encoding: A Framework for DNA Communication

**DOI:** 10.1371/journal.pone.0152774

**Published:** 2016-04-06

**Authors:** Bijan Zakeri, Peter A. Carr, Timothy K. Lu

**Affiliations:** 1 Department of Electrical Engineering and Computer Science, Department of Biological Engineering, Research Laboratory of Electronics, Massachusetts Institute of Technology, 77 Massachusetts Avenue, Cambridge, MA 02139, United States of America; 2 MIT Synthetic Biology Center, 500 Technology Square, Cambridge, MA 02139, United States of America; 3 MIT Lincoln Laboratory, 244 Wood Street, Lexington, MA 02420, United States of America; Universität Stuttgart, GERMANY

## Abstract

Synthetic DNA has great propensity for efficiently and stably storing non-biological information. With DNA writing and reading technologies rapidly advancing, new applications for synthetic DNA are emerging in data storage and communication. Traditionally, DNA communication has focused on the encoding and transfer of complete sets of information. Here, we explore the use of DNA for the communication of short messages that are fragmented across multiple distinct DNA molecules. We identified three pivotal points in a communication—data encoding, data transfer & data extraction—and developed novel tools to enable communication via molecules of DNA. To address data encoding, we designed DNA-based individualized keyboards (iKeys) to convert plaintext into DNA, while reducing the occurrence of DNA homopolymers to improve synthesis and sequencing processes. To address data transfer, we implemented a secret-sharing system—Multiplexed Sequence Encoding (MuSE)—that conceals messages between multiple distinct DNA molecules, requiring a combination key to reveal messages. To address data extraction, we achieved the first instance of chromatogram patterning through multiplexed sequencing, thereby enabling a new method for data extraction. We envision these approaches will enable more widespread communication of information via DNA.

## Introduction

Communication has many faces. While the general objective of transferring information between different parties remains constant, the medium for information transfer continues to evolve. In nature, DNA has been used for billions of years as the chemical of choice for transferring information across cells, species, and generations [1]. Now, advances in biotechnology are enabling the use of DNA for the transfer and storage of non-biological information [2–9].

Rapid advances in digital technologies over the past decades have enabled efficient and facile communication regardless of whether our messages are short tweets of 140 characters or long communiqués of thousands of pages. However, our ever-increasing reliance on digital technologies may make it worthwhile to explore new methods of information storage and transfer with alternative characteristics. Key attributes of DNA including high-density data storage, static and stable data maintenance, efficient reproducibility, and the lack of technological obsolescence, mean that DNA has distinct advantages over current magnetic and optical data storage platforms [1], thus warranting further exploration into DNA-specific writing and reading technologies.

**Fig 1:**
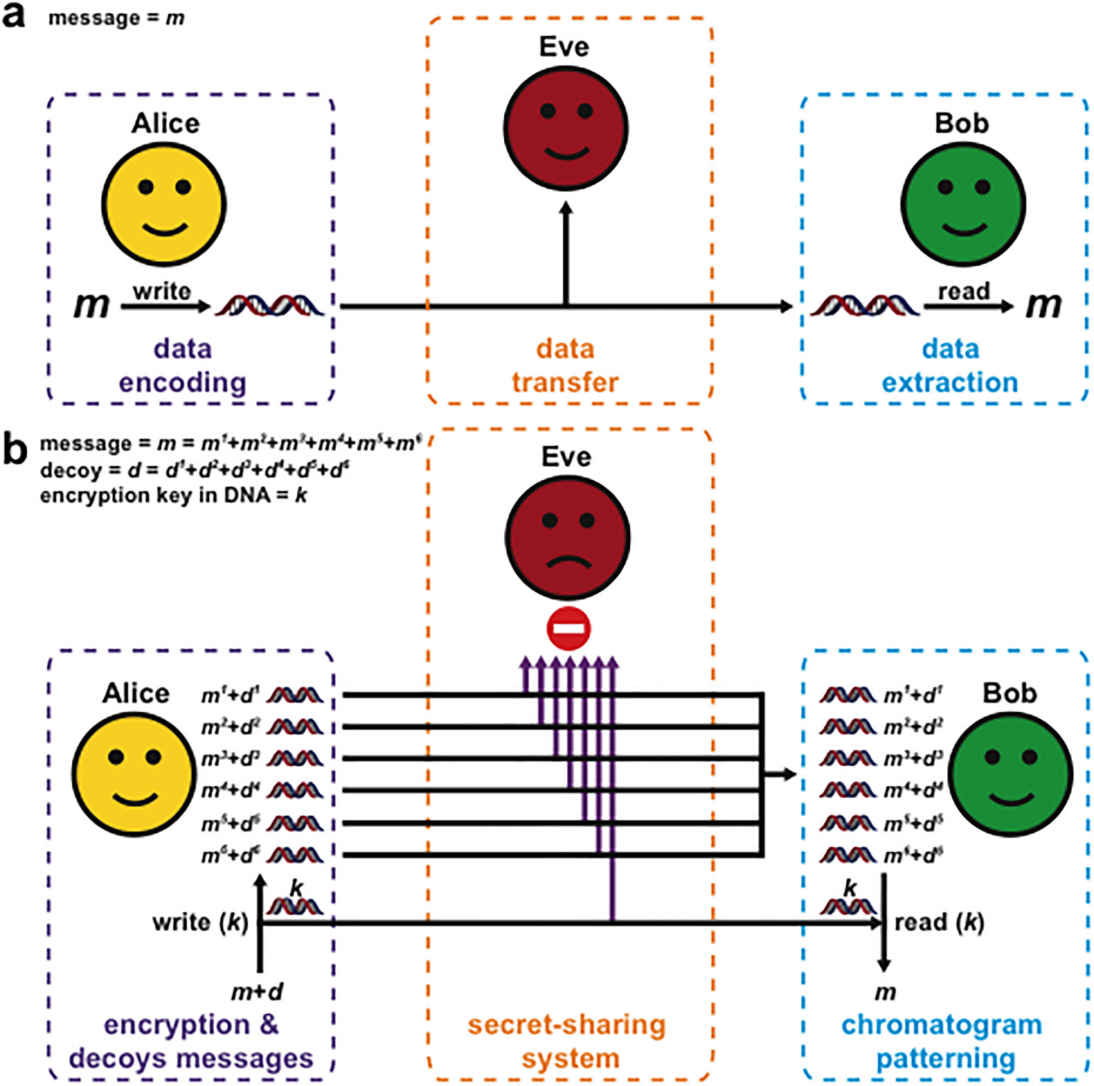
DNA communication. (a) For Alice to send a message (*m*) to Bob, she must first write the data into DNA and then physically send the DNA to Bob, who can read the DNA and extract the data. Eve, who is eavesdropping, can physically intercept and read *m*. Here we have identified three areas to explore within the communication channel between Alice and Bob: data encoding, data transfer, and data extraction. (b) Fragmented DNA communication. Data encoding: *m* can be mixed with decoy (*d*) data and fragmented, then written into DNA, where the key (*k*) is used to encode the data and can itself be written in DNA. Data transfer: DNA encoded *k* and fragmented *m*+*d* components can be transmitted between Alice and Bob using multiple different channels based on a secret-sharing system. Data extraction: chromatogram patterning can be used by Bob to extract data via multiplexed sequencing reactions.

With synthesis and sequencing speeds rising, and costs rapidly declining [10,11], DNA is an intriguing option for the transfer and storage of digital information [1]. DNA molecules have been used for hiding messages [2] and storing digital data [7–9]. In these studies, an encoding algorithm was used to convert digital data into nucleotide sequences that were then written, transferred, and read using DNA as the storage medium. The encoding algorithms were also programmed to reduce homopolymeric stretches as bits and trits were converted to bases. These methods allow users without prior biological or computer programming skills to encode any computer file into DNA sequences that can then be synthesized by commercial vendors. Therefore, we identified these three pivotal points of a communication—data encoding, data transfer & data extraction—to develop new methods for DNA-based communications (Fig 1a). To illustrate, if Alice sends a message (*m*) to Bob, she would first write—encode and synthesize—the information in DNA molecules and send it to Bob who would then read—sequence and decode—the message (*m*). However, during the transfer of *m* between Alice and Bob, Eve could intercept the communication and read *m*. To protect the information stored in *m*, DNA-specific cryptography and steganography methods may be implemented [12–15], akin to conventional digital data transfer that incorporate encryption algorithms such as AES, RSA, Twofish, and others. However, in these early days of DNA data storage and communication, we believe it to be also useful to explore different methods of data encoding, data transfer, and data extraction to find additional opportunities afforded by DNA compared to conventional magnetic and optical platforms.

Here we present a new framework for the communication of short messages in DNA that explores the fragmentation of a message across multiple distinct DNA molecules (Fig 1b). To encode a message *(m*) in DNA, we used an encoding key (*k*) to convert plaintext into bases, while at the same time reducing the occurrence of homopolymers. *k* was designed as a substitution cipher that assigned ‘codons’ to characters based on the frequency of occurrence of characters in English text. Additionally, decoy messages (*d*) were also encoded using *k* and incorporated within a communication. To decode the information, a combination key is required to discern the message *m* from the decoy information *d*, where subsequently *k* can be used to decode *m*. To transfer data, we established a secret-sharing system where *m* can be fragmented across a mixture of different DNA molecules, requiring Eve to physically intercept and interrogate multiple separate data transmission lines to gain access to *m*. To facilitate data extraction, we investigated a new method that allows for the multiplexed sequencing of multiple DNA molecules with a common primer, where regions within distinct DNA molecules that have matching information can be identified from a single sequencing reaction via chromatogram patterning.

## Materials and Methods

### Plasmids

Constructs were cloned using standard molecular biology techniques. KOD Hot Start DNA Polymerase (VWR) was used to PCR amplify a p15A origin and a chloramphenicol resistance gene, which were then fused to gBlocks from IDT (Table 1) using Gibson assembly. Random DNA sequences were generated at http://www.bioinformatics.org/sms2/random_dna.html. Constructs were sequence verified by Genewiz Inc. (Cambridge, MA).

**Table 1:** Sequences of the constructs used in this study.

Construct	Plasmid	Sequence
iKey-64	pBZ38	TTTTTTTTTTCGGAGCTGAGACCGAACGTAGGCTTCGGCACTGTTAGAAGATATCAACAATTCACGTATGCGCGTGGTAACTTGTCTTTTGATTCACTGCCATTCTGCGGAGCTCCCATTCAGATCCACCTGGAGGGGAAAGATAGTTTATGTCACACAGTACTAACAAAAACCCGGGTTTAGTCTAGGCGGTCCTGCCCCGTTTTTTTTTT
DNA-1	pBZ27	TGGCCACGATCCATGCTAACGTCTCTGCGTAGGGATGAATCCCGTTTTGAACTCGTTCCTACTGACGGACGAGCTGATAGGTAGCCGAAGTAGTGATACGATCCACACATGCCATCATTGCATACTCGTGCATTCAATGATGCATAGTCACGTAGTCCATATGGTAATGGTGATGTCAAGTCACATGTCAATACTCGTCACTAGAACTGAGCGCGATGACTGGCGAGCTGGTGCGCTCCCGAGGCTGGTCGAGCGACTAAGTTGAATGCGCAGACCGATCGAGACGACTCTAGCGCTGGAATAAATCAGAATAAAGA
DNA-2	pBZ28	CCCACCAATACTGCCAATAGACGGTACTGTACACCCTGTTTTACAGCAACGGGAAAGGAGGATCACTTTCTACAATTGTGTGCTGGACTGACAGTCGCATATCCACACATGCCATCATTGCATACTCGTGCATTCAATGATGCATCTACACGTAGTCCATATGGTAATGGTGATGTCACTACACATGTCAATACTCGTCACTAGAACTGAGCGCGATACGACTCGCCCATAGGGTTCGCCGGCTCGCACTGACTACCTTACGCTCTGACCCAGATCGGAGCCGGCCGCATGACCCCTGTGATATAATACCGTTCATC
n1	pBZ29	TAATACGACTCACTATAGGGACAGTCTAGTGCAGCAGTCAGTACGAGTCTCATGAGTGTAGGATGCATGATCATGATTCTGATCTAGTCCAGCAGTAGAGTCGTCTCGATCGATCTGTGCATCGTCAGCGATATTCGACGTAGTCGCTCGACCTGACTCGTGAGTGCAGCTACGTGTCAGTCATCCACTGTTGCCATATATGCAGACGGCATAGTATGCGTGTATGCGTCGAGAGATCATCCAGTTCTTGACGTTAGTTACAAGATTGGCCACGATCCATGCTAACGTCTCTTCCACCTTTCCCAAAAAGTAACACCGACTGATCGCGCATACGGCAACAGTGACTCTCGACTACCATAGTAGTGAGATGGTGGATTACGATCGCGTGATCTGAGTATCATTGATCTATAGTGGATTGACTGATGATCGTACTGTCGTACTGACTCTGACGTCGATCTCAGGTCATATTACTCGACAGTTGCTAAGTCAGTCATCGTCATACGATGCCGCTGAGCAATAACTAGC
n2	pBZ30	GCTAGTTATTGCTCAGCGGCATCGTATGACGATGACTGACTTAGCAACTGTCGAGTAATATGACCTGAGAGCTACTGATCTGACTAGCTAAGCTTGCATGCACGTCATGATCCACTATAGATCAATGATACTCAGATCACGCGATATCGACGTTGACTAGTCAAGCTAGATCCACATATGCTGTATGTGCGTAGTCGATGTCATGACTATGTTTTACAGCAACGGGAAAGGAGGACCGTCTATTGGCAGTATTGGTGGGATCTTGTAACTAACGTCAAGATAGGGATGATCTCTCGACGCATACACGCATTAGATGCCGTCTGCATATATGGCAACAGTGGATACGACTCGATCATCGAGTTCGCATGCTAGCACTGACTACGTTACGCTCTGATCTCAGACGATAGTCAGATCGGAGTCAGCTGCATGACGACAGTGCGATGCTAGCGTTGATCTCATGCATCCTACACTCATGAGACTCGTACTGACTGCTGCACTAGACTGTCCCTATAGTGAGTCGTATTA
n3	pBZ31	TAATACGACTCACTATAGGGACAGTCTAGTGCAGCAGTCAGTACGAGTCTCATGAGTGTAGGATGCATGATCATGATTCTGATCTAGTCCAGCAGTAGAGTCGTCTCGATCGATCTGTGCATCGTCGACGATATTCGACGTAGTCGCTCGACCTGACTCGTGAGTGCAGCTACGTGTCAGTCATCCACTGTTGCCATATATGCAGACGGCATAGTATGCGTGTATGCGTCGAGAGATCATCCAGTTCTTGACGTTAGTTACAAGATTGGCCACGATCCATGCTAACGTCTCTTCCACCTTTCCCAAAAAGTAACACACCATGACGTATCGACTACGCACATACAGCATATGTGGATGATCACTGACTGACTGAACTACGATCATGGTGTATGTGAGCGTGTATGTGCTCGTGACTGGAGAAACGGCAACAGTGGATGATTGACGTACGACTGCTAGCTCAGGTCATATTACTCGACAGTTGCTAAGTCAGTCATCGTCATACGATGCCGCTGAGCAATAACTAGC
n4	pBZ32	GCTAGTTATTGCTCAGCGGCATCGTATGACGATGACTGACTTAGCAACTGTCGAGTAATATGACCTGAGAGTCAGTGCTCATGATGTCAATCCACTGTTGCCGTTTCTCCCTACACGAGCACATACACGCTCACATACACCATGATGACTAGCATGATCATCCACCGTGTATCTAGATCACGCCGGCATGATCTGATGACGATCATGACTGTTTTACAGCAACGGGAAAGGAGGACCGTCTATTGGCAGTATTGGTGGGATCTTGTAACTAACGTCAAGATAGGGATGATCTCTCGACGCATACACGCATTAGATGCCGTCTGCATATATGGCAACAGTGGATACGACTCGATCATCGAGTTCGCATGCTAGCACTGACTACGTTACGCTCTGATCTCGGACGATAGTCAGATCGGAGTCAGCTGCATGACGACAGTGCGATGCTAGCGTTGATCTCATGCATCCTACACTCATGAGACTCGTACTGACTGCTGCACTAGACTGTCCCTATAGTGAGTCGTATTA
n5	pBZ33	TAATACGACTCACTATAGGGACAGTCTAGTGCAGCAGTCAGTACGAGTCTCATGAGTGTAGGATGCATGATCATGATTCTGATCTAGTCCAGCAGTAGAGTCGTCTCGATCGATCTGTGCATCGTCACGGATATTCGACGTAGTCGCTCGACCTGACTCGTGAGTGCAGCTACGTGTCAGTCATCCACTGTTGCCATATATGCAGACGGCATAGTATGCGTGTATGCGTCGAGAGATCATCCAGTTCTTGACGTTAGTTACAAGATTGGCCACGATCCATGCTAACGTCTCTTCCACCTTTCCCAAAAAGTAACACTGACTGCATTCGTGATCATCATGCCGGCGTGATCTAGATACACGGTGGATTCAGCTACTAGTCGAATCATGACGTGAGAAGCATGAACGATATGAAGAAGTTATGTGGATAGCTGTCGACGTGATCGTATCGATGCAGTCCTCAGGTCATATTACTCGACAGTTGCTAAGTCAGTCATCGTCATACGATGCCGCTGAGCAATAACTAGC
n6	pBZ37	GCTAGTTATTGCTCAGCGGCATCGTATGACGATGACTGACTTAGCAACTGTCGAGTAATATGACCTGAGAGCTATCGATGACGTACTGATGTCATCATGATCCACATAACTTCTTCATATCGTTCATGCTTCTCACGTCATGATAACGCATCCACCATCTCACTACTATGGTAGTCGAGCTACACTGTTGCCGTATGCGCGATGTCAATTGTTTTACAGCAACGGGAAAGGAGGACCGTCTATTGGCAGTATTGGTGGGATCTTGTAACTAACGTCAAGATAGGGATGATCTCTCGACGCATACACGCATTAGATGCCGTCTGCATATATGGCAACAGTGGATACGACTCGATCATCGAGTTCGCATGCTAGCACTGACTACGTTACGCTCTGATCCTAGACGATAGTCAGATCGGAGTCAGCTGCATGACGACAGTGCGATGCTAGCGTTGATCTCATGCATCCTACACTCATGAGACTCGTACTGACTGCTGCACTAGACTGTCCCTATAGTGAGTCGTATTA

### Sanger Sequencing

Constructs were purified using Qiagen kits and stored in cell culture grade water (Cellgro). Constructs were diluted to 30 ng/μL and sent for sequencing at indicated ratios. Primer_ExternalFw_ (GACATTAACCTATAAAAATAGGC), Primer_ExternalRv_ (GCATCTTCCAGGAAATCTC), Primer_Key_ (TAATACGACTCACTATAGGG), and Primer_Message_ (GCTAGTTATTGCTCAGCGG) were used for Sanger sequencing reactions performed in triplicate at Genewiz Inc. under ‘Difficult Template’ settings. Genewiz Inc. was not consulted prior, during, or after this study and all Sanger sequencing reactions were performed under blind conditions to ensure bias was not introduced in the results.

### Next-Generation Sequencing

An outside party (MIT BioMicro Center, Cambridge, MA) performed next-generation sequencing (NGS) sequencing and analysis on a mixture of n1+n2+n3+n4+n5+n6. Plasmids were purified using Qiagen kits and stored in cell culture grade water (Cellgro). To confirm purity, plasmids (300ng) were run on a 1% agarose gel. Plasmids were then mixed at equal concentrations of 30 ng/μL and 900 ng of the mixture was submitted to the MIT BioMicro Center. Blind experimental conditions were used throughout the sequencing and annotation process.

Briefly, for NGS sequencing a Nextera kit (Epicentre) followed by 1.5% agarose BluePippin (Sage Science) isolation of 450–800 bp inserts was used to generate a library. A MiSeq (Illumina) run on a 600 nt v3 kit was used for pair-end sequencing. Sequence assemblies where then performed using various programs including: SOAP Denovo, Trinity, Mira, Velvet, and RAST annotation.

## Results and Discussion

To date, several elegant methods have been proposed for encoding digital information in DNA, each taking a unique approach to convert digital data into bases while at the same time reducing the occurrence of homopolymeric stretches [16]. However, within these early days of the field different encoding methods need to be investigated and the pros and cons of different approaches evaluated until the field converges on a single standardized and DNA-centric encoding method.

**Fig 2:**
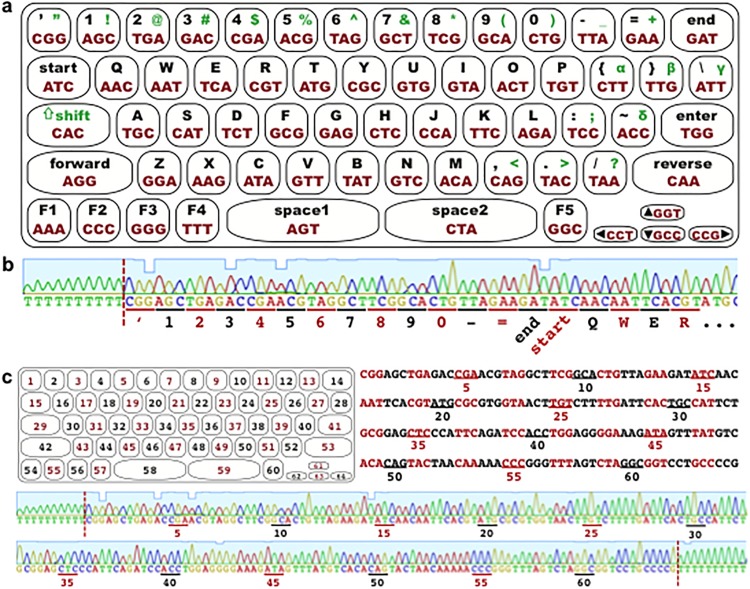
64 button iKey for chromatogram patterning. (a) iKey-64, used to convert plaintext to codons for writing information in DNA molecules. Messages begin with ‘start’, finish with ‘end’, ‘forward’ and ‘reverse’ provide information on the strand containing the desired message, and ‘space1’ and ‘space2’ may be used to produce troughs in chromatograms. The ‘shift’ codon precedes capitalized letters or upper characters. Codons can be randomized to produce up to 24!12!28! = 9.1x10^61^ iKey-64 variants. (b) The iKey-64 variant from (a) written in synthetic DNA and read by Sanger sequencing. Shown is the first row and part of the second row of the iKey keyboard (flanked by 10 T nucleotides). (c) Top: iKey-64 buttons and codons were numbered to write the keyboard onto a strand of DNA. Bottom: iKey-64 written in DNA. Codons were flanked by 10 Ts to separate the start and end of the keyboard from surrounding DNA for identification, marked by red lines.

To convert plaintext to bases for DNA encoding, we took inspiration from written text. We combined the familiarity of text-based communication—the QWERTY keyboard—and the genetic code to develop individualized keyboards (iKeys) that serve as a facile method for DNA communication. The natural genetic code employs three-letter DNA words (codons) to represent the 20 common amino acids used to build proteins. The four-letter DNA alphabet of adenine (A), cytosine (C), guanine (G) and thymine (T) thus yields 4^3^ = 64 distinct codons. Accordingly, codons are units of nucleotides that encode information that is then translated into function. Here, we abstract the concept of a codon to encode information by mapping the 64 distinct codons onto a modified QWERTY keyboard to produce a personalized code—iKey-64—for translating text into DNA (Fig 2a). This serves as an encoding key (*k*) for converting a message (*m*) into a DNA encodable language (Fig 1b), akin to a substitution cipher. Furthermore, any specific version of iKey-64 can itself be encoded in DNA and provided as an additional component of a communication, serving as a unique dictionary for each message (Fig 2b and 2c).

**Fig 3:**
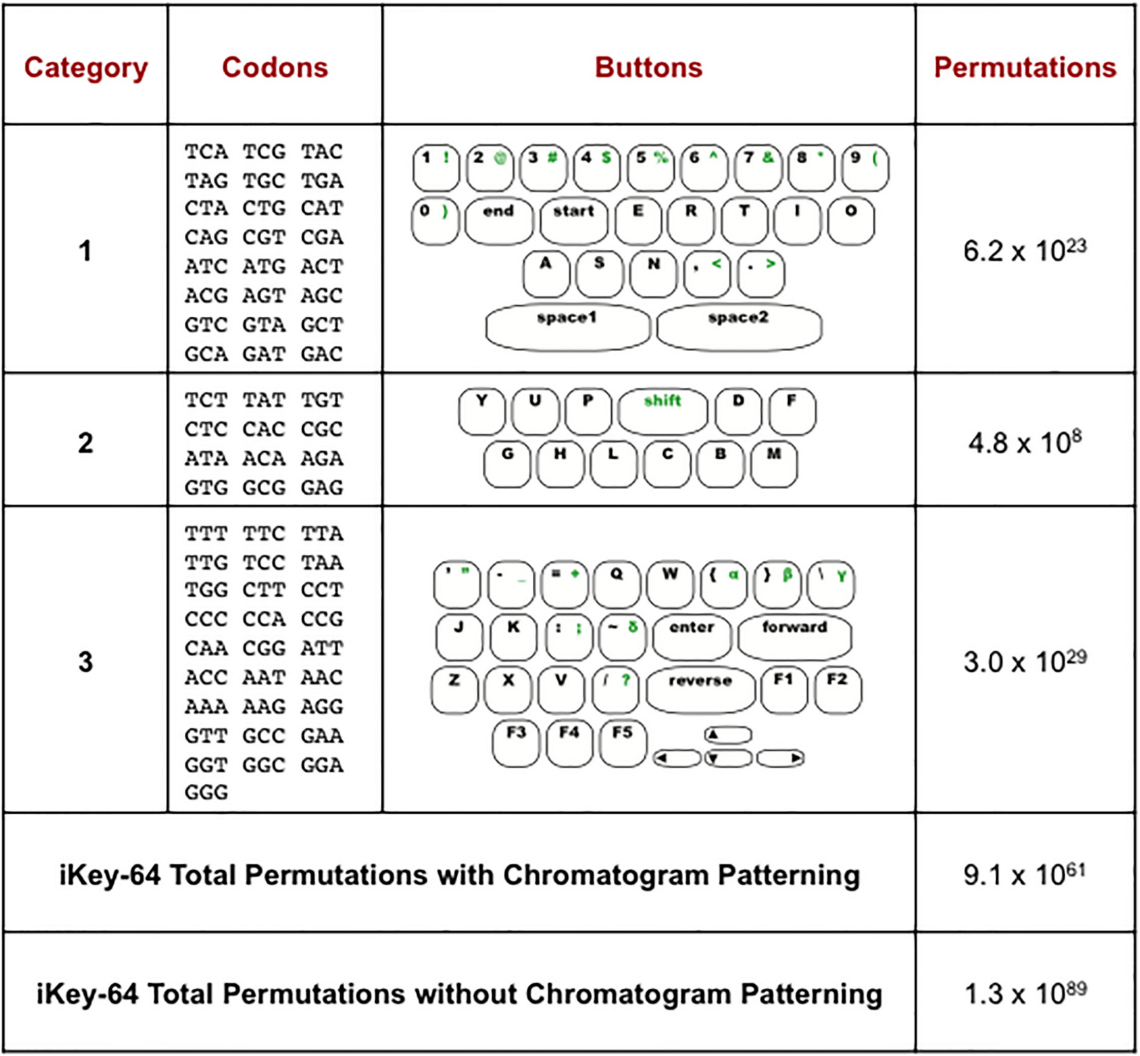
Rational design of iKey-64 for encoding information into DNA, while reducing the incidence of homopolymers and achieving chromatogram patterning. The buttons of iKey-64 were separated into 3 categories based on the frequency of use as judged by qualitative measures. Category 1 is for the most frequently used buttons and is encoded by codons that contain three different nucleotides. Category 2 is for less frequently used buttons and is encoded by codons that contain the same nucleotide in the first and third position. Category 3 is for the least frequently used buttons and is encoded by codons that contain two or more homopolymers.

It is known that stretches of homopolymers in DNA often lead to sequencing inaccuracies [16]. To mitigate this problem, we rationally designed iKey-64 to reduce the incidence of homopolymers in DNA messages by basing codon assignment on the frequency of use of letters in the English language [17] (Table 2 and Fig 3). Higher frequency characters were designated to codons containing 3 different nucleotides, lower frequency characters to codons with the same nucleotide in the first and last position, and the least frequent characters were assigned to codons with 2 or more homopolymeric stretches. Here we use English as an example, but a similar approach can be used for other languages. Since the codons AAA, CCC, GGG, and TTT are assigned to function keys—that can encode any user-defined function—no homopolymeric stretches longer that 4 bases are possible when encoding regular English text (Fig 2a). For example, the letter VK would be encoded with bases GTTTTC, where the maximum homopolymeric stretch of 4 Ts would be reached. Additionally, since all numerals (0–9) were assigned to codons containing 3 different nucleotides, no homopolymeric stretches longer than 2 bases are possible when encoding numbers, including instances where digital data stored in bits, trits, etc. is converted to bases (Fig 2a). For example, the numerals 110011 would be encoded with bases AGCAGCCTGCTGAGCAGC, where the maximum homopolymeric stretch of 2 Cs would be reached. In the event where multiple consecutive function keys are used, spaces can be used to reduce homopolymeric stretches. In subsequent experiments we investigate new methods for fragmented DNA communication. Therefore, we encode text as an example since the contents of the communication are not our focus, but similar approaches should be applicable for other data formats.

**Table 2:** The frequency of letters used in English based on the Concise Oxford Dictionary and adapted from [17].

Letter	Frequency	Letter	Frequency
E	11.16%	M	3.01%
A	8.50%	H	3.00%
R	7.58%	G	2.47%
I	7.54%	B	2.07%
O	7.16%	F	1.81%
T	6.95%	Y	1.78%
N	6.65%	W	1.29%
S	5.74%	K	1.10%
L	5.49%	V	1.01%
C	4.54%	X	0.29%
U	3.63%	Z	0.27%
D	3.38%	J	0.20%
P	3.17%	Q	0.20%

To investigate this DNA platform for information transfer, we sought to disseminate texts across multiple different DNA strands so that the desired message would be revealed only if the correct strand combinations were analyzed. A single communication channel between Alice and Bob can be intercepted by Eve at a single point of contact, thereby compromising the message *m* (Fig 1a). However, a fragmented communication channel would require multiple points of contact for interception by Eve (Fig 1b). This approach can add an additional layer of protection for a communication and also provide opportunities to explore introducing tiers of complexity within a communication that is afforded by the unique makeup of DNA as a chemical polymer for information storage. Therefore, we created a fragmented communication platform that we call Multiplexed Sequence Encoding (MuSE), a secret-sharing system [18] that allows for communication of a message *m* across multiple distinct DNA molecules.

To extract information that is fragmented by MuSE across multiple distinct DNA molecules, one would have to sequence the DNA molecules individually then compare the sequences to look for regions of sequence identity to locate encoded messages. However, the distinct nature of DNA as a data storage medium provided us with an opportunity to explore alternative methods of data extraction. Accordingly, we sought to develop a platform that allows for multiple distinct DNA molecules to be sequenced within a single reaction, whereby the encoded data shared among DNA molecules could be easily located via patterns formed in sequencing chromatograms.

**Fig 4:**
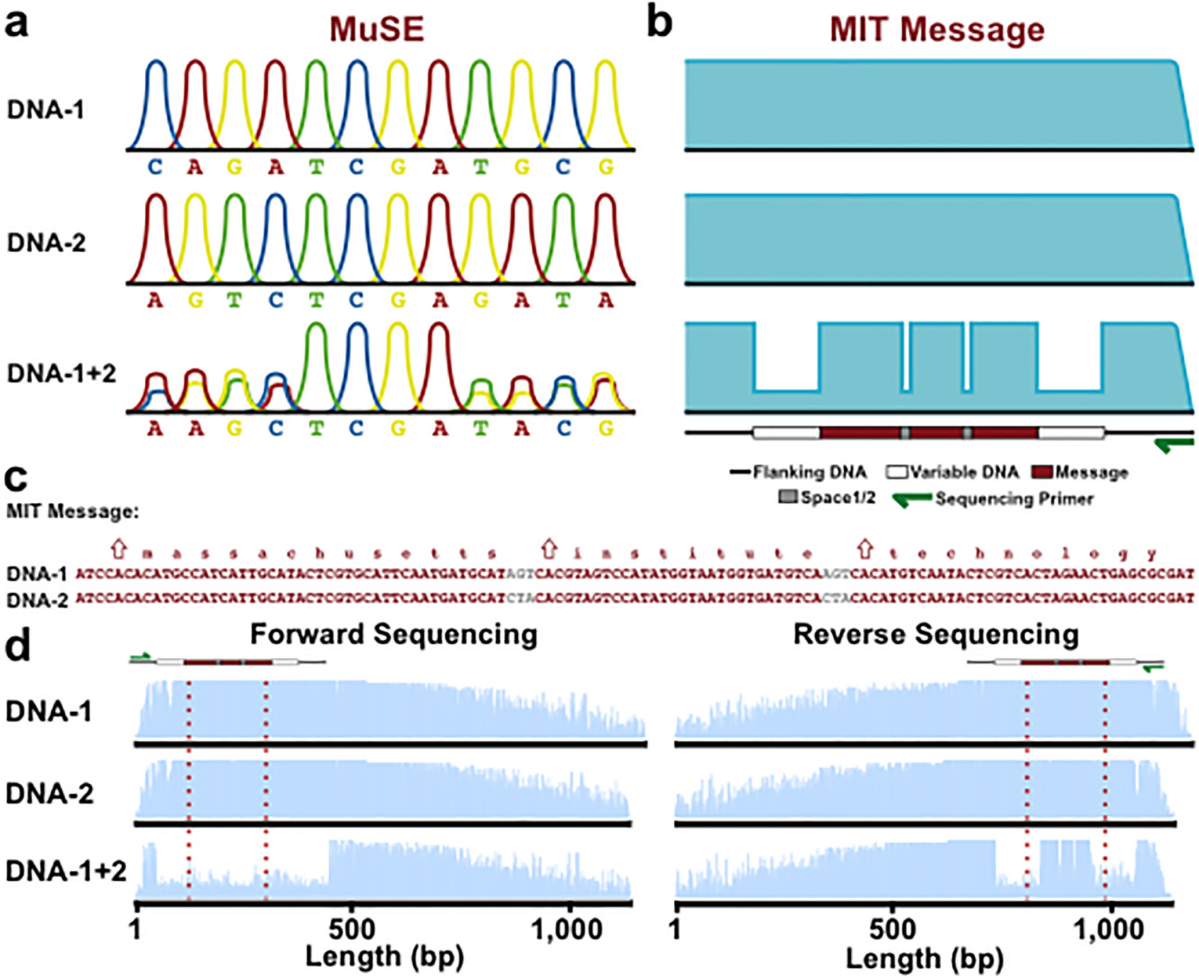
Chromatogram patterning with MuSE. (a) Schematic for chromatogram patterning. When two DNA strands are co-sequenced with a common primer via Sanger sequencing, different overlapping nucleotides produce small heterogeneous peaks while matching nucleotides produce large homogeneous peaks. Peaks are kept in alignment via iKey-64. (b) Schematic of chromatogram patterning for the message ‘Massachusetts Institute Technology’ via MuSE. (c) Sequences for ‘Massachusetts Institute Technology’ used in (b) and encoded with iKey-64. (d) Chromatograms observed from Sanger sequencing of the DNA-encoded message described in (b) and (c). When DNA-1 and DNA-2 are co-sequenced at equal concentrations with a common primer (green arrows), chromatogram patterning is achieved during reverse (Primer_ExternalRv_) but not forward (Primer_ExternalFw_) sequencing due to the flanking variable DNA regions. Red lines surround embedded messages.

**Fig 5:**
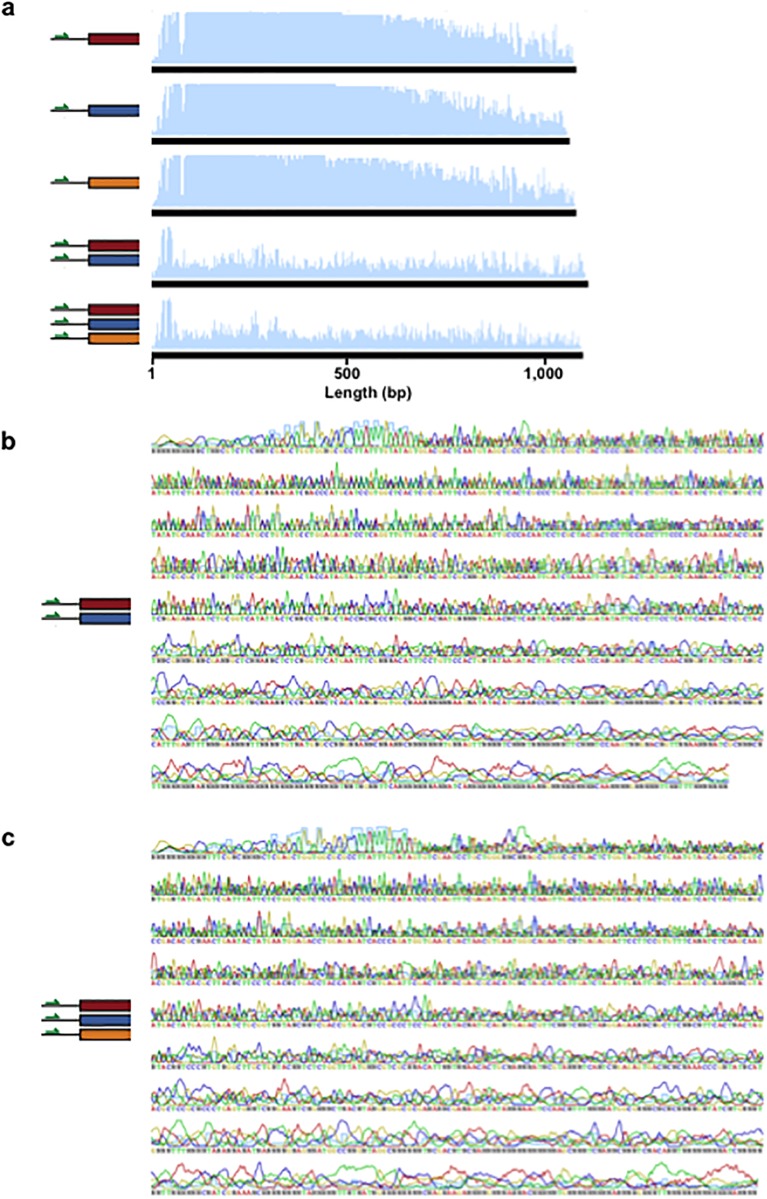
Naïve co-sequencing of multiple DNA strands. (a) Red (DNA-1), blue (n1), and orange (iKey-64) strands have different sequences but they all share a common upstream region and sequencing primer (Primer_ExternalFw_). Individual sequencing of each strand produces high quality reads, but the resulting reads are of poor quality when two (red and blue) or three (red, blue, and orange) strands are co-sequenced. (b) Close-up of the chromatogram of red and blue co-sequencing. (c) Close-up of the chromatogram of red, blue, and orange co-sequencing.

In designing MuSE, we expected that when multiple DNA strands are analyzed together by Sanger sequencing using a common primer, at chromatogram positions where two bases are identical a large homogeneous peak would be observed, and where two bases differ a small heterogeneous peak would be observed, thereby producing a pattern (Fig 4a). Not surprisingly, the naïve sequencing of multiple DNA strands with a common primer is unable to achieve chromatogram patterning, and instead it produces poor readouts (Fig 5). However, the codons in iKey-64 were rationally assigned to characters based on the frequency of use of individual characters, thereby serving to reduce the incidence of homopolymers in DNA messages that reduce the accuracy of sequencing reactions. Therefore, we expected the design of iKey-64 to mitigate the problem of base calls moving out of phase when multiple DNA molecules were sequenced simultaneously with a common primer as observed in Fig 5.

**Fig 6:**
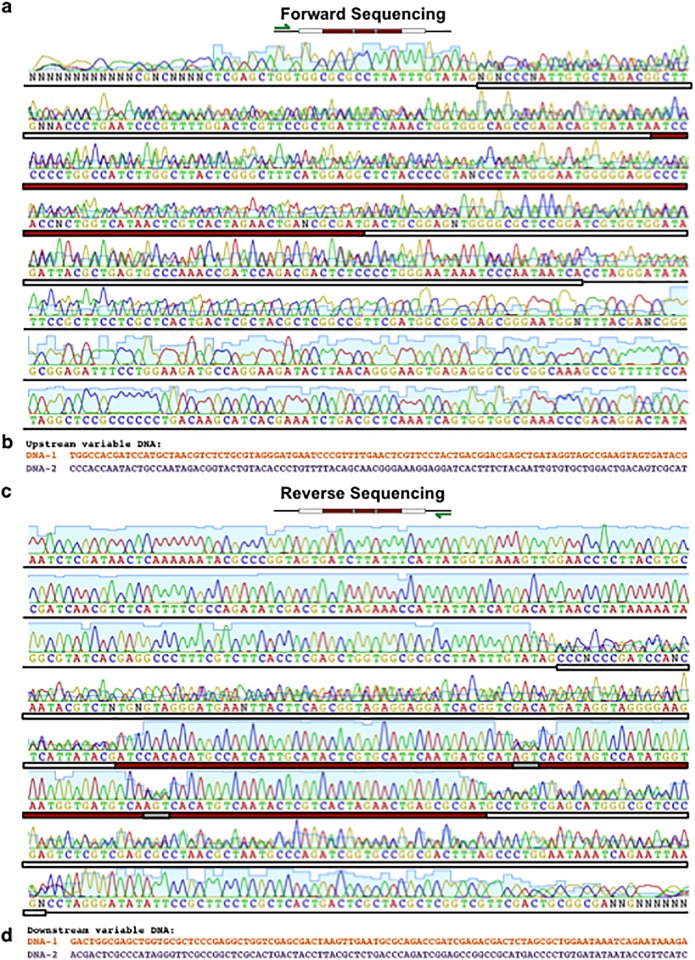
Chromatogram patterning requires the alignment of base calls to be maintained during co-sequencing of DNA strands. (a) Close-up of the chromatogram for forward co-sequencing of DNA-1+2 encoding the MIT message (red box) from Fig 4d. (b) Sequence of the upstream variable DNA regions (Fig 4b), corresponding to the upstream flanking region of the MIT message. (c) Close-up of the chromatogram for reverse co-sequencing of DNA-1+2 encoding the MIT message (red box) from Fig 4d. (d) Sequence of the downstream variable DNA regions (Fig 4b), corresponding to the downstream flanking region of the MIT message. Samples were co-sequenced at equal concentrations and the green arrows depict the sequencing primers (Primer_ExternalFw_ and Primer_ExternalRv_).

To test whether chromatogram patterning could be achieved with MuSE, we used iKey-64 to encode the message ‘Massachusetts Institute Technology’ on two DNA strands, where space1 (AGT) was used with the first DNA strand (DNA-1) and space2 (CTA) with the second DNA strand (DNA-2) to demarcate individual words in the sequences (Fig 4b and 4c). In this design, co-sequencing both DNA strands together should introduce troughs around words in the resulting chromatogram, thereby providing a simple method to locate the message from a single sequencing reaction. As expected, individual sequencing of DNA-1 and DNA-2 produced high quality reads, but gave no indication of the presence or location of a message (Fig 4d). However, in a DNA-1+2 mixture, forward sequencing with a common primer did not reveal a message through chromatogram patterning, but rather camouflaged the message (Fig 4d). This was due to variable DNA sequences placed upstream of the messages, where stretches of C and A homopolymers at the 5’ ends interfered with base determination during Sanger sequencing, thus causing intentional misalignment of the recognized bases in the chromatogram (Fig 6a and 6b). Only reverse sequencing of DNA-1+2 with a common primer produced a distinct pattern in the chromatogram, readily identifying the location of the message to be decoded with iKey-64 (Fig 4d). Since there were no interfering stretches of homopolymers in the variable DNA regions, there were no shifts in the base calls during sequencing, thus leading to predictable chromatogram patterning from a multiplexed sequencing reaction (Fig 6c and 6d). Therefore, as a proof-of-concept we demonstrated that information from multiple DNA molecules can be extracted in a single reaction.

**Fig 7:**
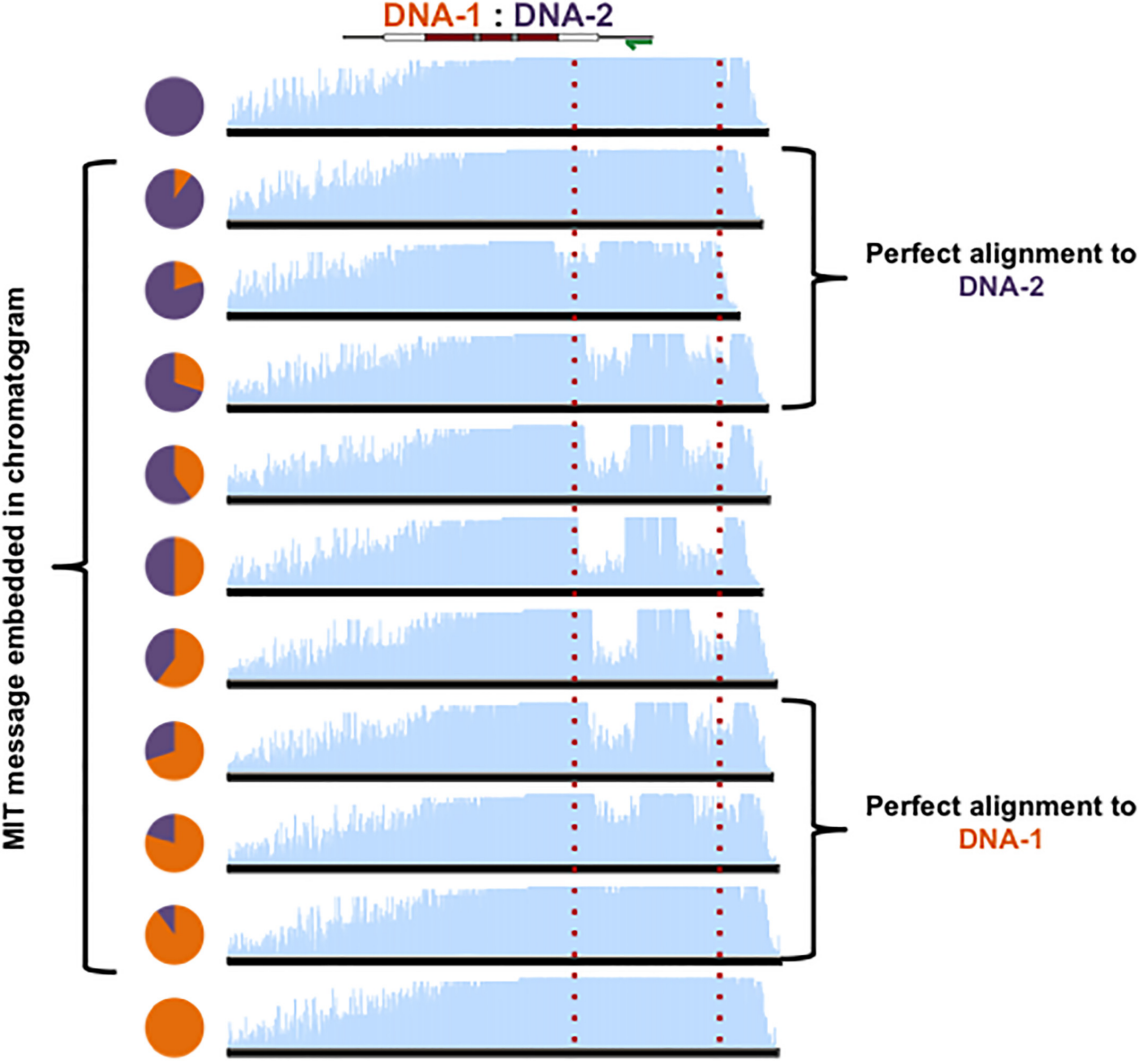
MuSE can be tuned to hide information in DNA communications. Chromatogram patterning can be tuned to discreetly embed information in sequencing data by varying the ratios of DNA-1 (orange) and DNA-2 (purple). Red lines surround embedded messages.

**Fig 8:**
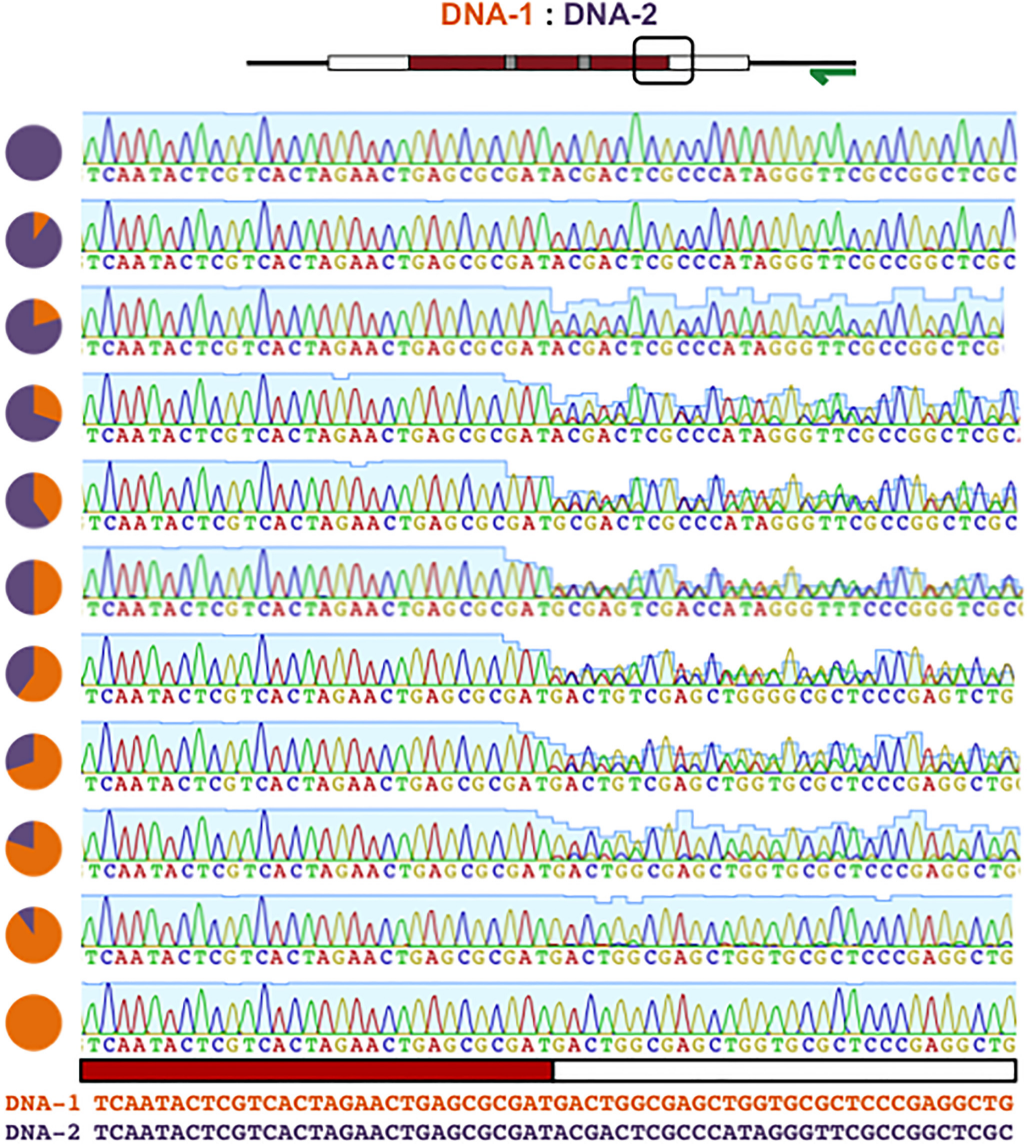
Discreet embedding of information in chromatograms. A close-up of chromatogram patterns formed with MuSE tuning from Fig 7. Message encoding regions (red box) contain single peaks while variable DNA regions (white box) contain two overlapping peaks whose heights can be adjusted by varying the ratios of DNA-1:DNA-2. The chromatogram close-ups correspond to the boxed region in the MIT message schematic shown above.

**Fig 9:**
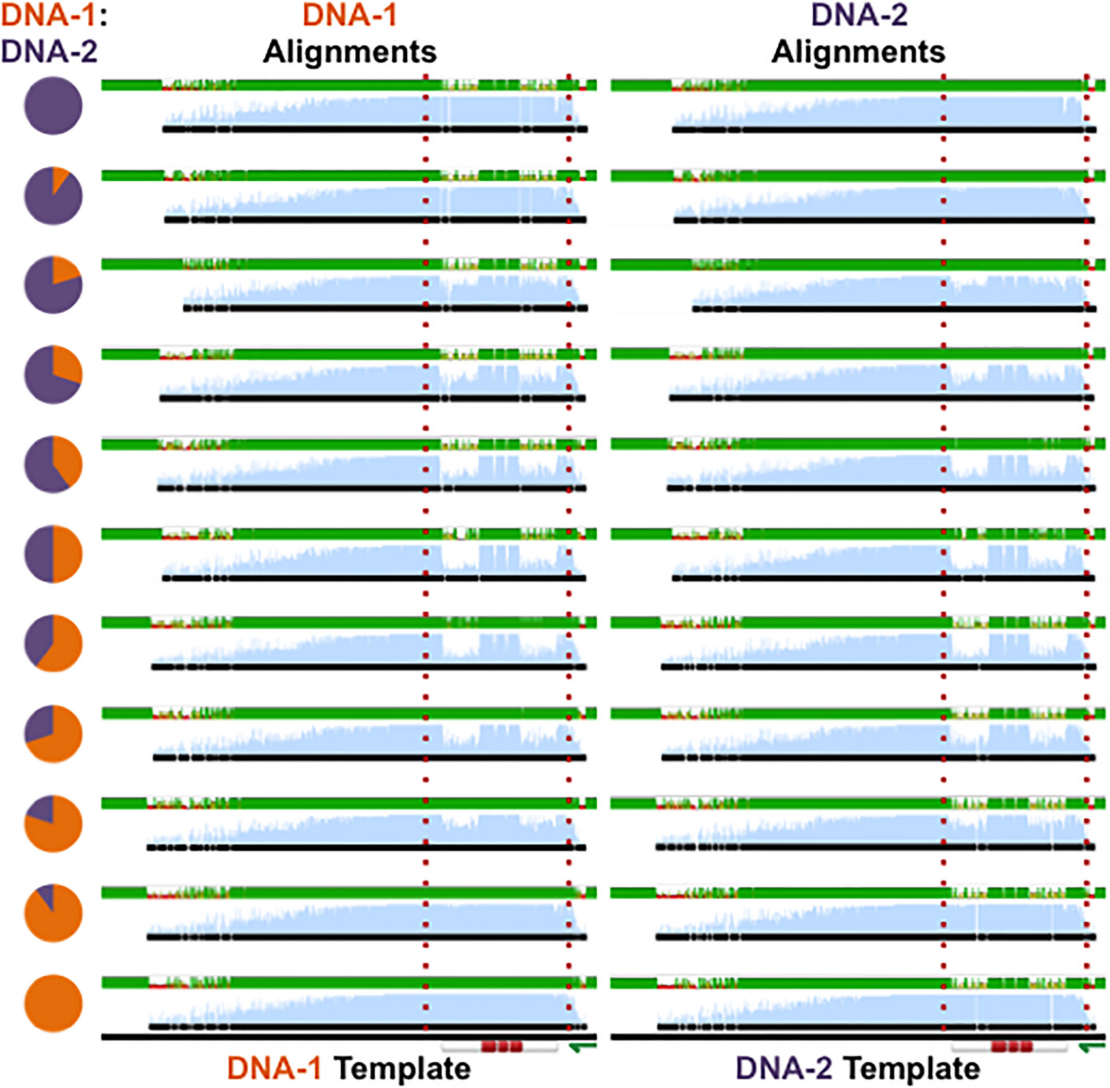
Discreetly embedded messages cannot be identified by sequence alignments. By varying the ratios of DNA-1 (orange) and DNA-2 (purple), the degree of chromatogram patterning can be tuned (Fig 7). When one partner is present at a lower concentration, chromatogram patterning is still achieved; however, the resulting chromatogram aligns perfectly with the more concentrated partner. Therefore, messages may be discreetly encoded between two DNA strands and revealed in chromatograms, but not identified by sequence alignments. Left: alignment of chromatograms from Fig 7 with DNA-1. Right: alignment of chromatograms from Fig 7 with DNA-2. Red lines surround embedded messages.

While individual sequencing of each strand followed by sequence alignments can be used to extract information from multiple DNA molecules, chromatogram patterning provides opportunities to explore new methods for data extraction and for incorporating information in DNA mixtures. To illustrate, the degree of contrast achieved in the chromatogram patterns can be tuned in a MuSE communication by adjusting the ratio of DNA-1/DNA-2 (Figs 7 and 8). This serves as a method to embed information in chromatograms discreetly so that alignments of DNA sequencing data to known templates cannot be used to identify embedded information (Fig 9). Such an approach provides new opportunities for exploring ways to store information in DNA, where data extraction is dependent on multiplexed DNA sequencing.

**Fig 10:**
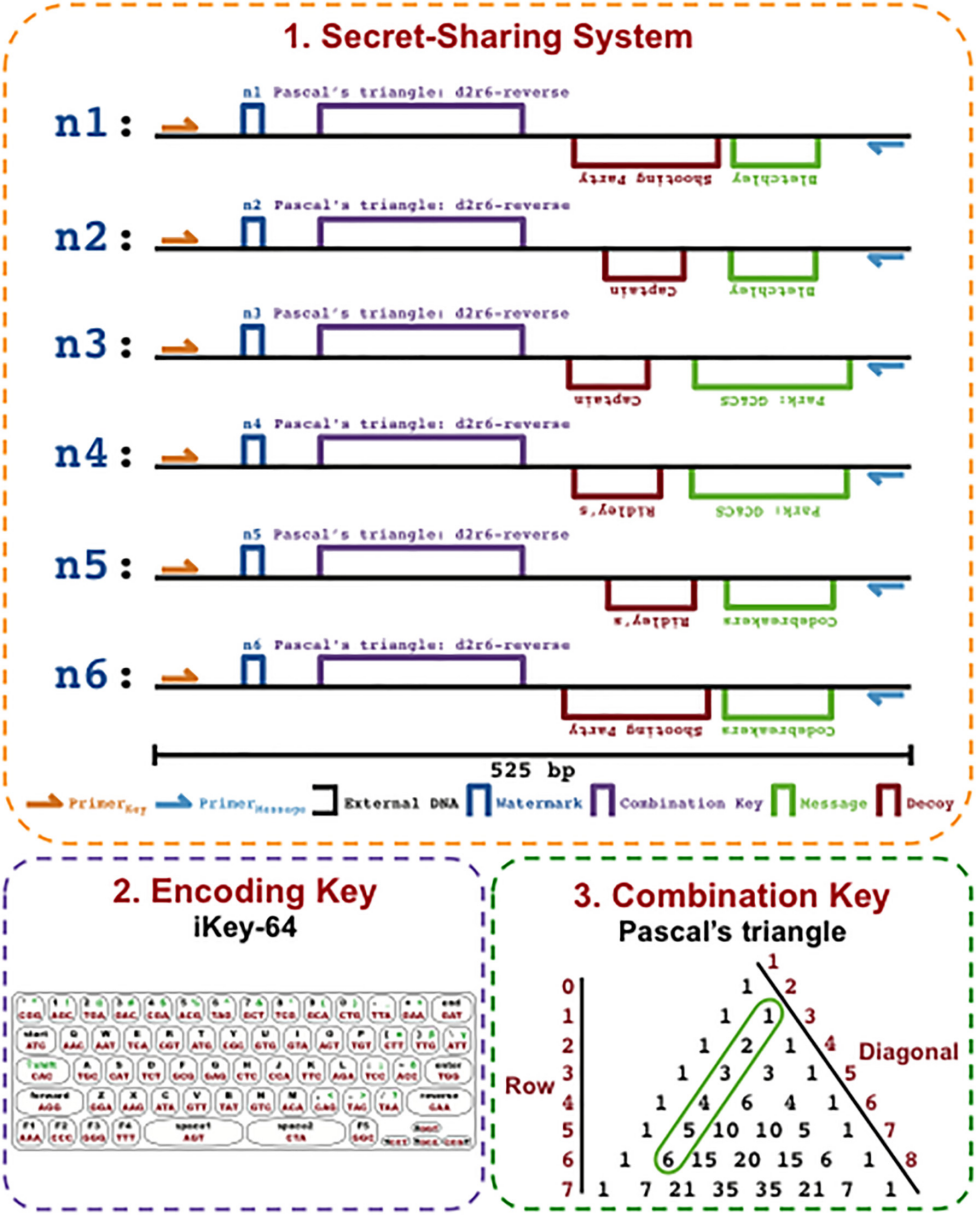
A fragmented WWII communication. 1. Secret-Sharing System: a recreated WWII communication was encoded across six DNA molecules and included watermarks, a combination key, a desired message, and a decoy message. 2. Encoding Key: iKey-64 was used to encode the information included in the WWII communication. 3. Combination Key: identifies which strands contain the desired message, here if strands are sequenced according to the Combination Key—obtained from Pascal’s triangle—with the appropriate primers, then the desired message is revealed.

Next we wanted to determine whether we could use the MuSE method to disseminate information encoded with iKey-64 across more than 2 DNA molecules. This would enable us to introduce more complexity into a fragmented communication channel (Fig 1b). To demonstrate, we sought to fragment a communication that contained an intended message and a decoy message across 6 distinct DNA molecules. Such a communication would include three components (Fig 10): (1) secret-sharing system: the intended message and the decoy message along with instructions on how to differentiate between the two would be disseminated across 6 DNA molecules, (2) encoding key: the information would be converted from plaintext into bases using iKey-64, and (3) combination key: a puzzle would enable the end-user to identify the strand combinations that need to be analyzed in order to extract the desired message.

**Fig 11:**
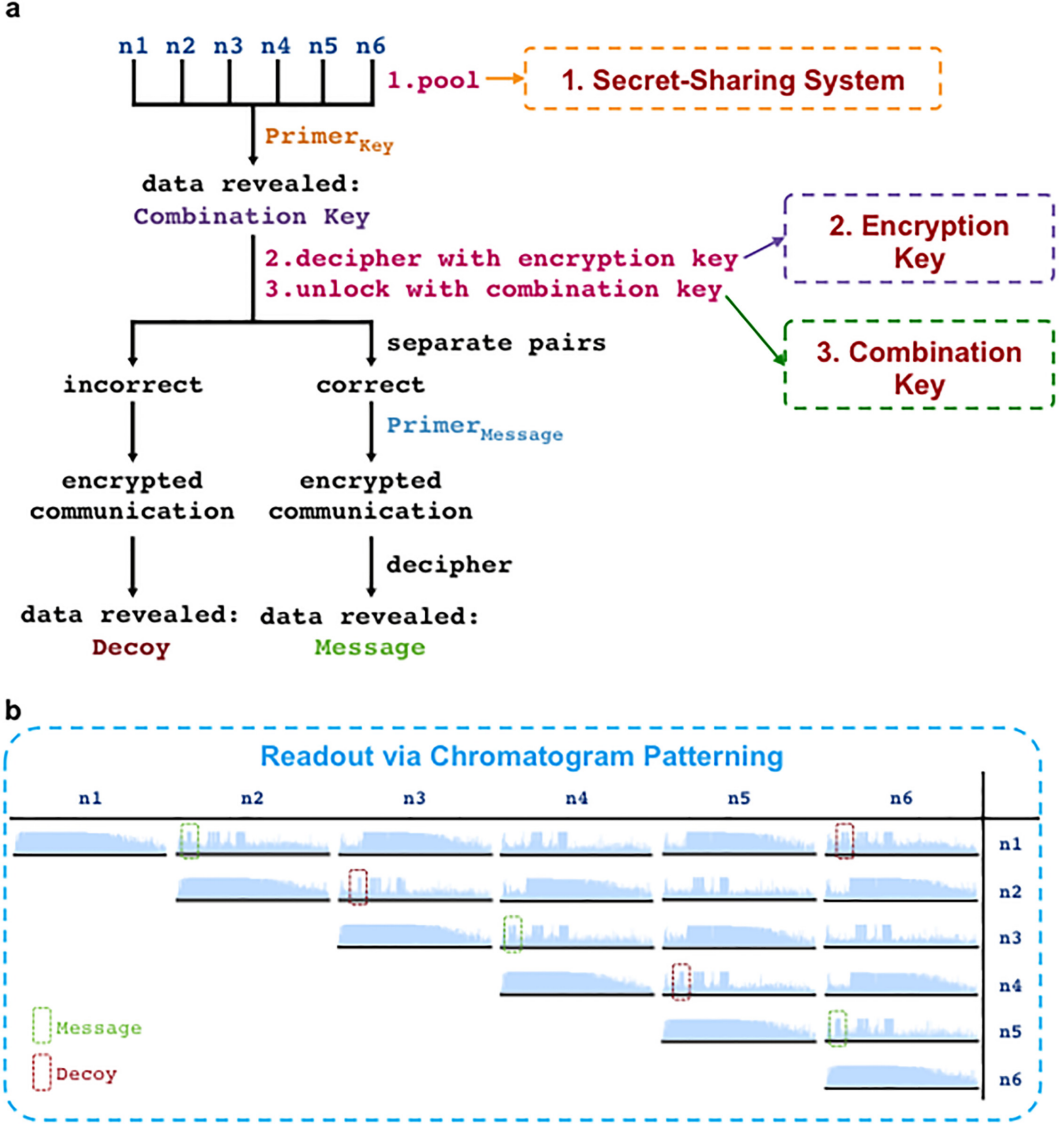
A WWII communication recreated in DNA. (a) Workflow for extracting the desired message from the WWII communication. Workflow steps 1, 2, and 3 are highlighted in pink and can be viewed in detail in Fig 10. Data containing strands are pooled and sequenced with Primer_Key_ to reveal the combination key. Decoding and solving the combination key will reveal the correct strand pairs to analyze with Primer_Message_ to reveal the desired message. Analysis of incorrect strand pairs will reveal a decoy message. (b) Chromatograms of an n1 x n6 matrix of DNA strands from the WWII communication (Fig 10) tuned and co-sequenced with Primer_Message_. Boxes highlight patterns that communicate either the desired message (green) or the decoy message (red).

Accordingly, iKey-64 was used to encode watermarks, a combination key, a desired message, and a decoy message within 525 bp regions across six synthetically produced DNA strands, recreating a World War II communication made during the establishment of Bletchley Park [19] (Figs 10 and 11a), a significant point in cryptography history. The functions of the elements are: (i) watermarks—an identification tag for each DNA strand that allows the end-user to categorize each strand according to the combination key, (ii) combination key—a riddle whose solution provides the correct combinations of DNA strands required to analyze in order to unlock the desired message, (iii) message—the desired information to be communicated, and (iv) decoy—a false message to be revealed if improper strand combinations are analyzed, for example as a result of an incorrect solution to the combination key.

A workflow of the process for the WWII communication encoded with iKey-64 is shown in Fig 11a to demonstrate how an end-user would extract information from our DNA communication. The first step would be to pool a partial sample of the available 6 DNA molecules (n1+n2+n3+n4+n5+n6) obtained from the fragmented DNA communication within the secret-sharing system. The next step is to identify the combination key in order to know which strand combinations need to be analyzed to reveal the desired message. Co-sequencing of the pooled DNA molecules with Primer_Key_, which is common to all 6 DNA molecules, followed by decoding with iKey-64 should reveal the information: “Pascal’s triangle: d2r6-reverse” (Fig 10). Here a simple combination key was chosen to demonstrate the concept, and this riddle means that the desired message is revealed from sequencing DNA pairs in the reverse direction as ordered in Pascal’s triangle from diagonal 2 down until row 6. Next, the desired message can be extracted by co-sequencing the correct DNA pairs using the sequencing primer Primer_Message_, which is common to all 6 DNA molecules. Thus, if strand pairs n1+n2, n3+n4, and n5+n6 were to be co-sequenced using Primer_Message_ and decoded with iKey-64, then the embedded message “Bletchley Park: GC&CS Codebreakers” would be revealed. If, for example, one were to misinterpret the key, then a decoy message would be revealed—“Captain Ridley’s Shooting Party”—as a result of co-sequencing DNA pairs n2+n3, n4+n5, and n6+n1, a circular permutation of the correct combination key.

**Fig 12:**
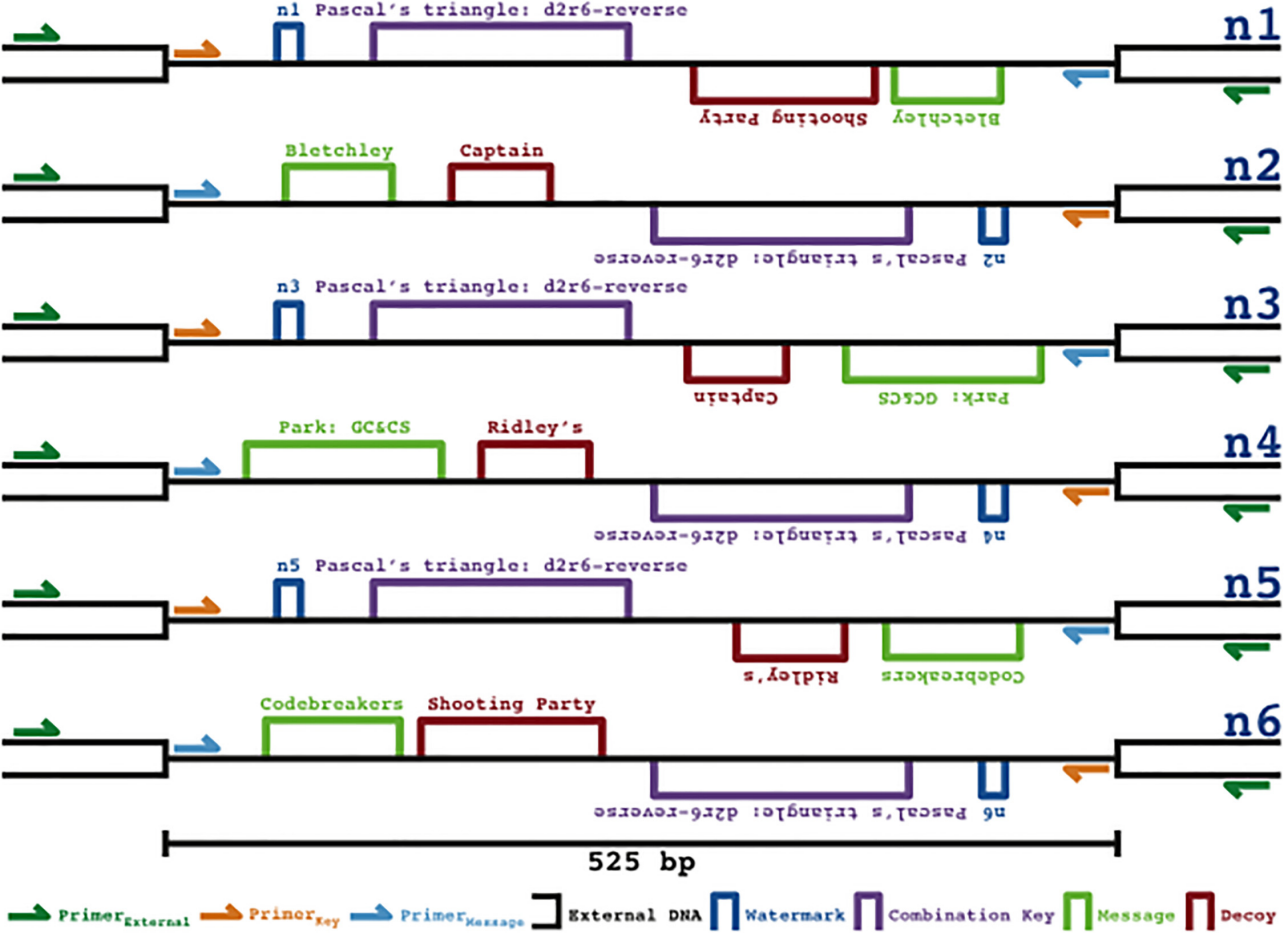
DNA camouflage. The 525 bp information-encoding regions of the WWII communication were flipped between the forward and reverse strands to provide a camouflage effect against sequencing with random primers (Primer_ExternalFw_ and Primer_ExternalRv_). While the external DNA regions surrounding the information containing regions were identical, strands n1/n3/n5 were placed in the forward direction and strands n2/n4/n6 in the reverse direction, with watermarks used to determine the orientation.

**Fig 13:**
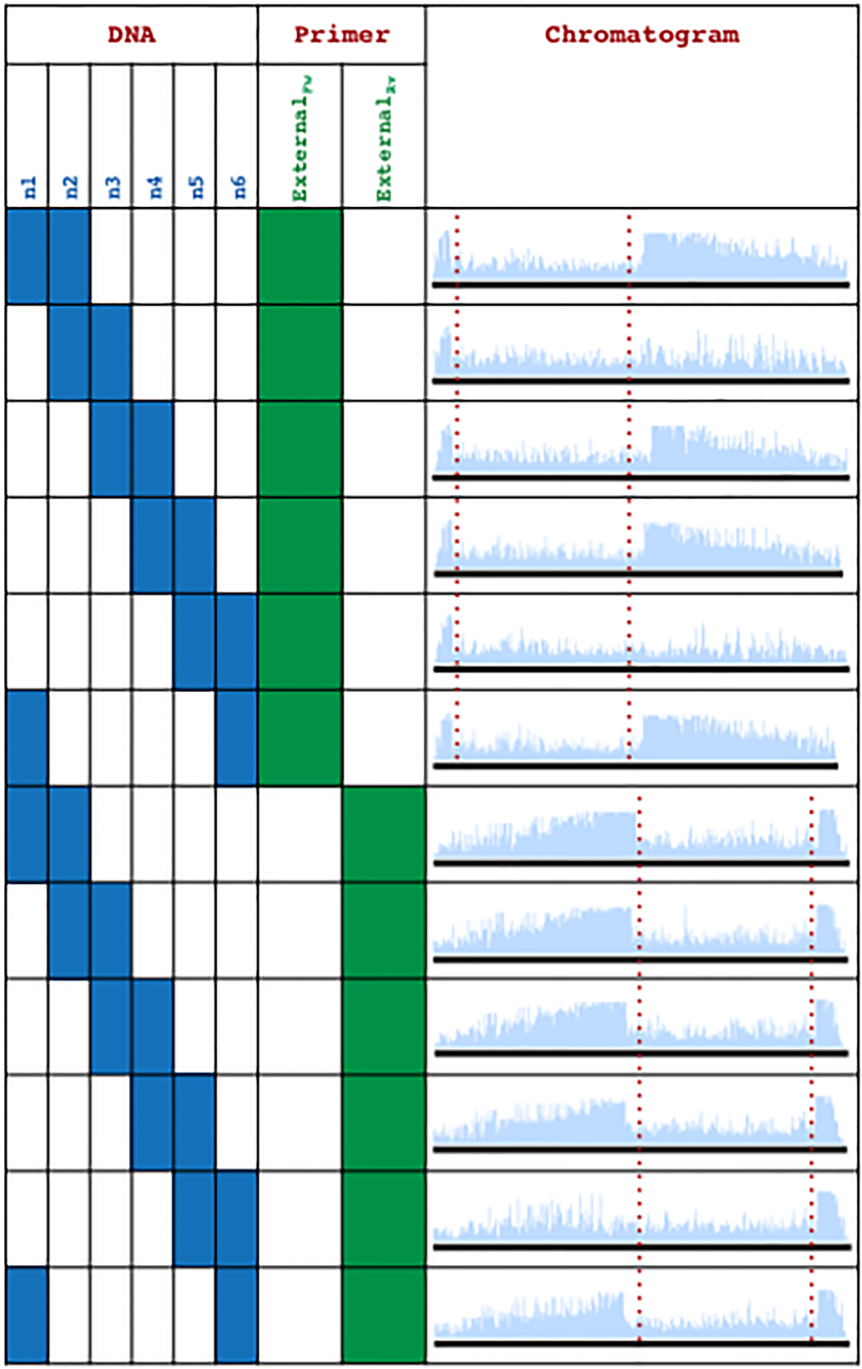
Data extraction from the WWII communication using Primer_ExternalFw_ and Primer_ExternalRv_ produces poor quality sequencing reads (message encoding regions are between the red lines).

**Fig 14:**
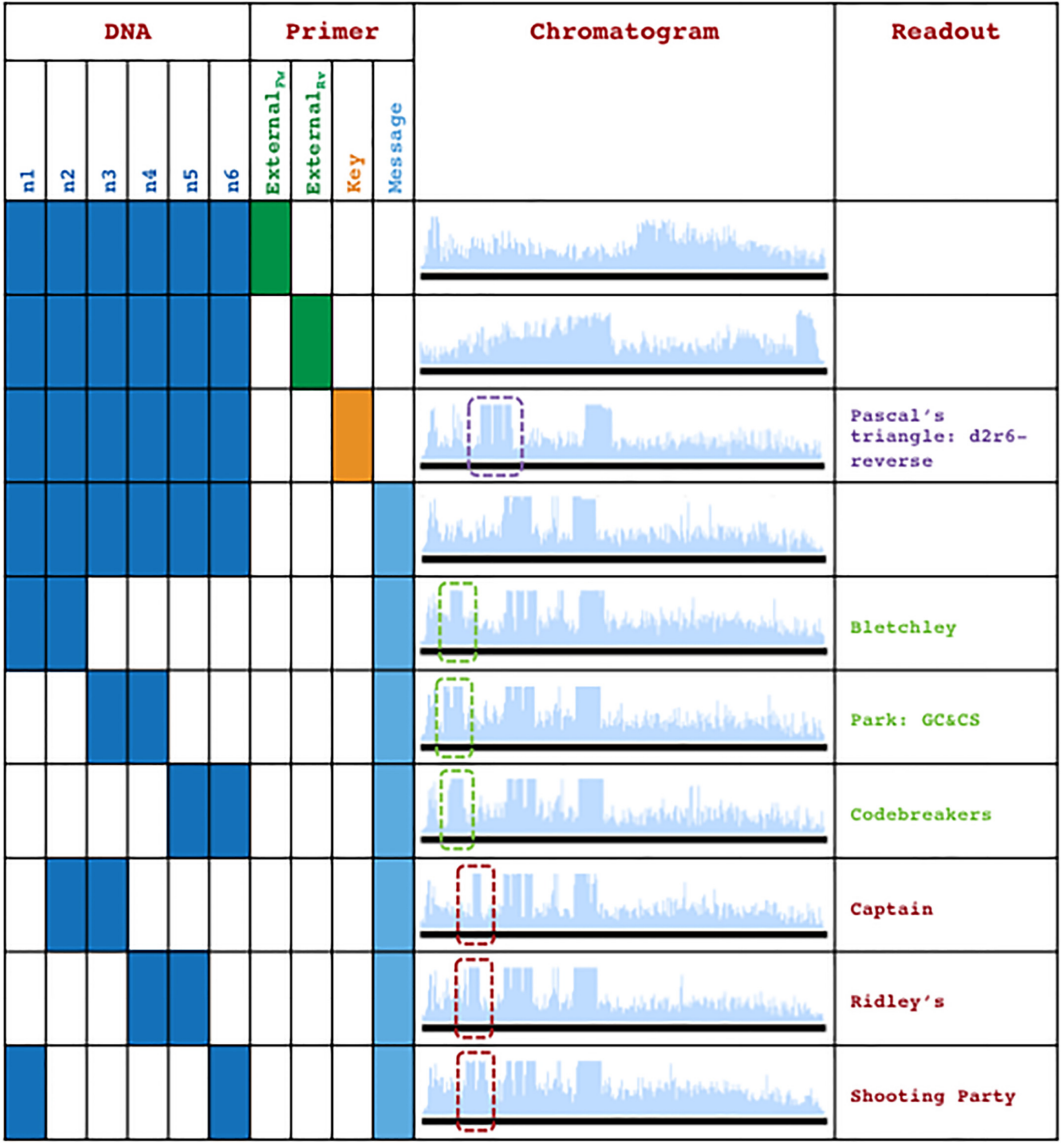
WWII communication readouts of tuned and co-sequenced DNA strands.

In the event that the end-user does not have access to Primer_Key_ and Primer_Message_—an unauthorized user such as Eve (Fig 1b)—then random sequencing primers may be used. For example, the sequencing primers Primer_ExternalFw_ or Primer_ExternalRv_ (Fig 12) may be used instead of Primer_Key_ and Primer_Message_ to extract messages embedded in DNA fragments. As a way to obfuscate random sequencing attempts of pooled DNA samples, we flipped the information-containing regions of our WWII communication between the forward and reverse strands. We hypothesized that this would create a camouflage effect, where co-sequencing reactions would not produce chromatogram patterning and instead produce sequencing reads of poor quality that did not provide reliable sequence information (Fig 12). As intended, co-sequencing with Primer_ExternalFw_ or Primer_ExternalRv_ did not produce chromatogram patterning, regardless of whether message or decoy pairs (Fig 13), or all six strands were co-sequenced (Fig 14).

**Fig 15:**
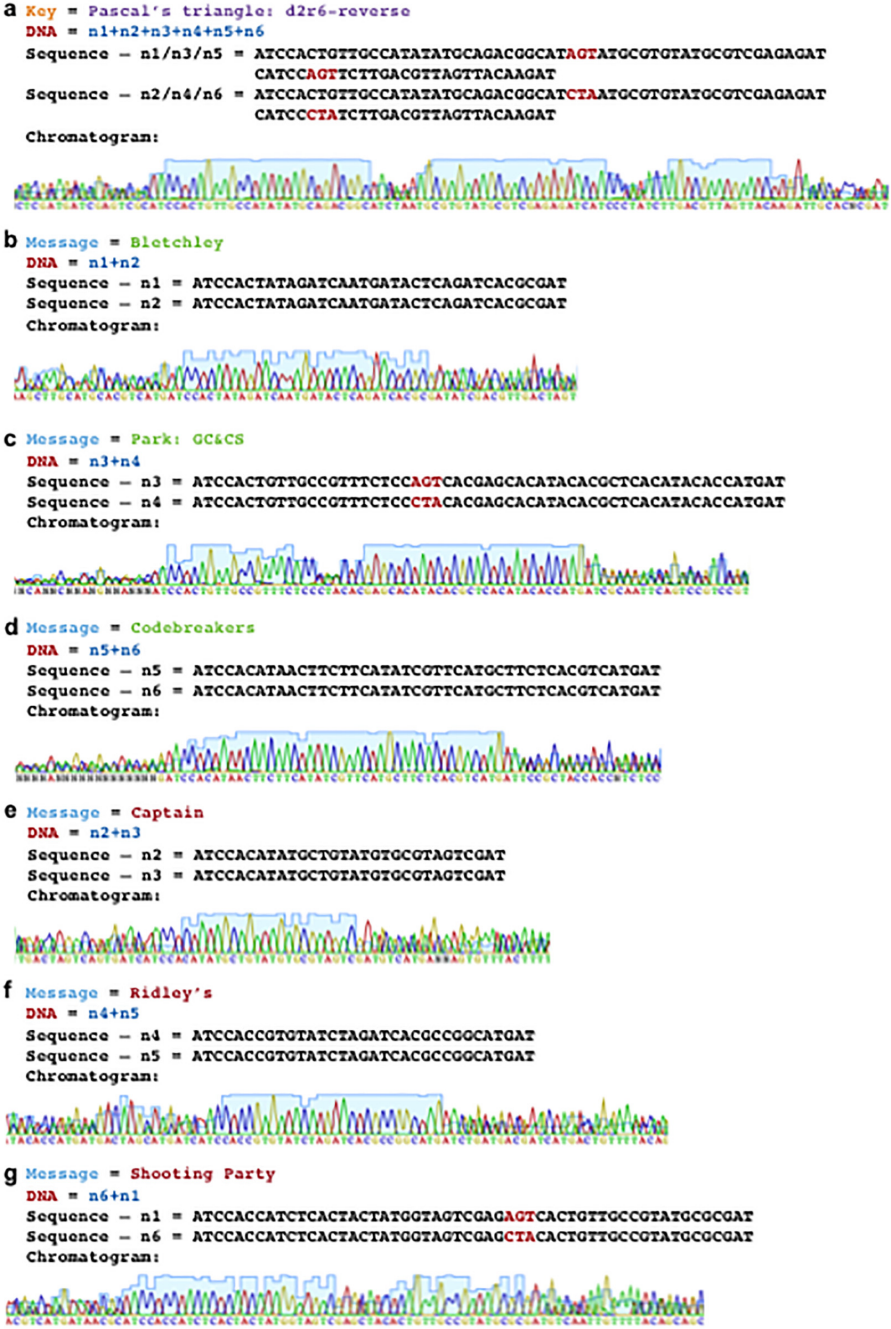
Examination of the peaks produced during co-sequencing of the WWII communication. Details of the encoded information, strand combinations tuned and co-sequenced, DNA sequence of embedded messages, and close-ups of the chromatogram patterns produced are shown for the WWII communication including: (a) the combination key, (b), (c), (d), the desired message, and (e), (f), (g), the decoy message. Space1 was used for all odd numbered strands (n1, n3, n5) and space2 was used for all even numbered strands (n2, n4, n6) to demarcate words. Space1/2 codons are shown in red.

On the other hand, if the appropriate sequencing primers are used as per the data extraction workflow (Fig 11a), then the information from the fragmented DNA communication can be efficiently extracted. To demonstrate, when Primer_Key_ is used to co-sequence a pooled sample of all six DNA molecules from the WWII communication (Fig 10), then the combination key “Pascal’s triangle: d2r6-reverse” is revealed via chromatogram patterning while the other data encoding regions (watermark, message, and decoy data) do not lead to chromatogram patterning (Fig 14). Similarly, chromatogram patterning is not observed as expected when Primer_Message_ is used for co-sequencing all six strands, since the proper strand combinations are not being co-sequenced as per the combination key. However, co-sequencing of DNA pairs with Primer_Message_ as per the order in Pascal’s triangle—n1+n2, n3+n4, and n5+n6—reveals the message “Bletchley Park: GC&CS Codebreakers” via chromatogram patterning (Fig 15). Alternatively, the co-sequencing of the incorrect pairs—n2+n3, n4+n5, and n6+n1—reveals the decoy message “Captain Ridley’s Shooting Party” (Fig 15). Expectedly, co-sequencing of other pair combinations did not lead to any patterning (Fig 11b).

**Fig 16:**
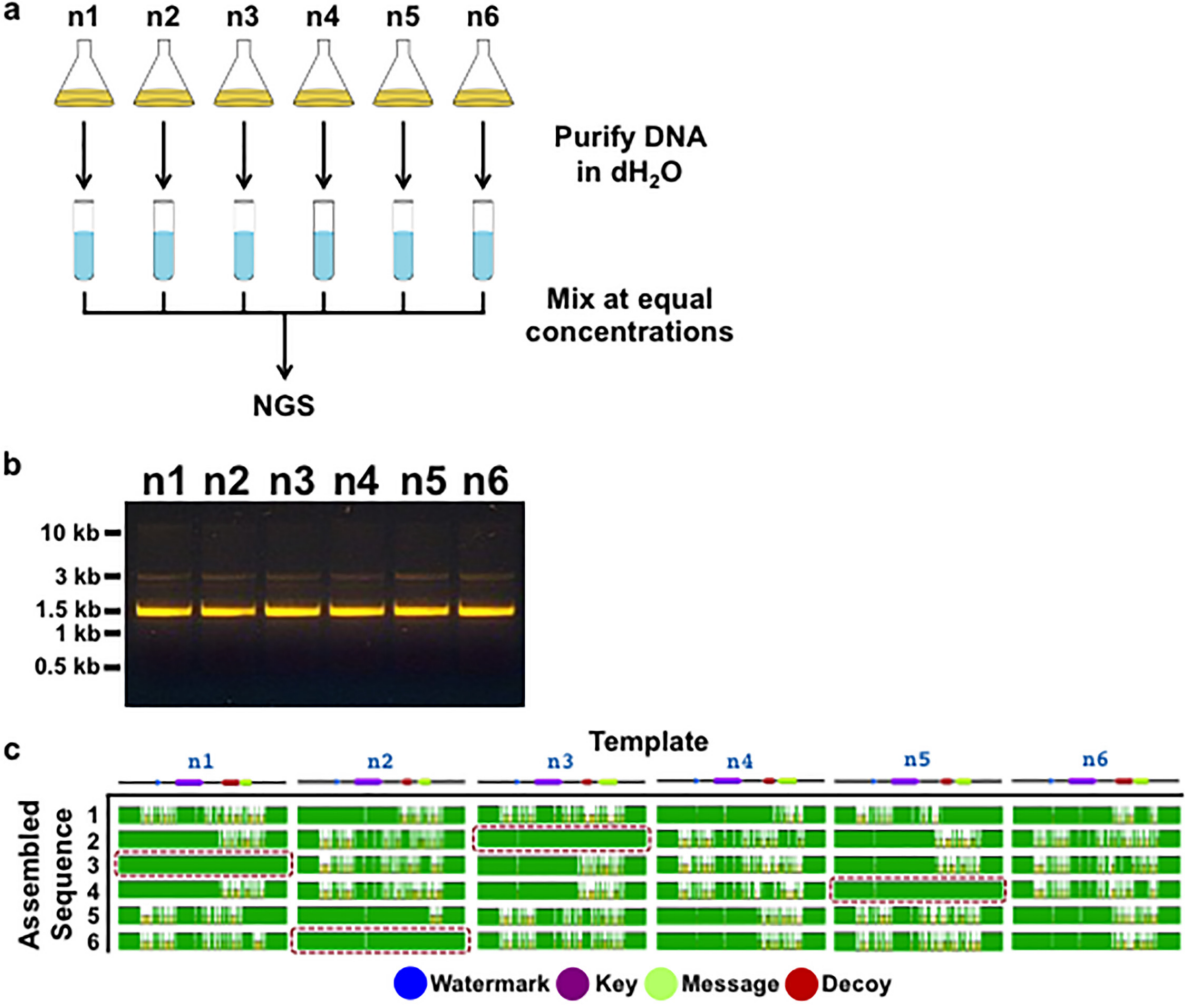
Next-generation sequencing of the WWII communication. (a) Plasmids containing n1, n2, n3, n4, n5, and n6 sequences (Fig 10) were grown and purified in dH_2_O, mixed at equal concentrations of 30 ng/μL, and submitted to an outside party (MIT BioMicro Center) for NGS sequencing and assembly under blind experimental conditions. (b) 300 ng of plasmids containing n1, n2, n3, n4, n5, and n6 sequences were run on a 1% agarose gel to demonstrate purity. (c) The outside party (MIT BioMicro Center) was provided with the number of plasmids, vector sequences, and the size of messages inserted into the vectors and asked to assemble the messages encoded in the plasmids. They assembled 6 sequences (Table 4) that represent the messages n1, n2, n3, n4, n5, and n6. Here the alignment of the 6 assembled sequences with n1, n2, n3, n4, n5, and n6 templates are shown. Shown below is a legend for the color-coding of the templates. Boxes highlight assembled sequences with near perfect alignment to corresponding templates.

While co-sequencing of a pooled DNA communication with primers that are not specific to the messages results in poor quality sequencing reads and a camouflage effect with Sanger sequencing (Fig 13), an unauthorized end-user may use other sequencing platforms such as next-generation sequencing (NGS) to gain access to encoded information. To recreate such a scenario, we tested the difficulty associated with NGS analysis of DNA samples, where the end-user has no prior knowledge of the DNA sequences or what is encoded within them. Accordingly, we prepared a pooled and purified DNA sample containing the DNA molecules n1+n2+n3+n4+n5+n6 from the WWII communication (Fig 10). We then submitted the sample for NGS analysis to an outside party under blind experimental conditions, asking them to provide us with the assembled contents of the sample (Fig 16a and 16b). While sequencing of the mixture produced ~2 million reads (Table 3), the blind assembly of the reads to reconstruct the contents proved difficult and inconclusive. However, after the initial analysis we informed the outside party that there were 6 plasmids in the sample, each containing 525 bp messages as inserts. We further provided the vector sequence and asked for the exact sequences of the messages in the sample. A second round of analysis identified 6 assembled sequences that represented our encoded information (Table 4). Alignment of the 6 identified sequences with n1, n2, n3, n4, n5, and n6 templates provided most of the information in the six DNA molecules, with n1, n2, n3, and n5 providing almost perfect sequence alignments (Fig 16c). Therefore, an end-user should be able to extract data from a fragmented communication using NGS with prior knowledge of the DNA contents and the encoding method.

**Table 3:** Next-generation sequencing statistics of assembled reads under blind experimental conditions.

	n1+n2+n3+n4+n5+n6*
**Sequence size**	1,407,947
**Number of scaffolds**	2,851
**% GC**	51.1
**Shortest contig size**	300
**Median sequence size**	423
**Mean sequence size**	493.8
**Longest contig size**	4,625
**Number of subsystems**	22
**Number of coding sequences**	984
**Number of RNAs**	0

*NGS sequencing of a mixture of samples n1+n2+n3+n4+n5+n6 (Fig 16) produced 1,997,179 reads at 300 bp with 47% GC content. Shown are the statistics of the assembled scaffolds by the MIT BioMicro Center under blind experimental conditions. While the DNA samples produced high quality reads, under blind experimental conditions assembly of the reads into the original constructs proved challenging and the results were inconclusive. Information regarding the plasmids containing the WWII communication: n1 = 2,346 bp/47.4% GC, n2 = 2,346 bp/47.3% GC, n3 = 2,346 bp/47.5% GC, n4 = 2,346 bp/47.6% GC, n5 = 2,346 bp/47.4% GC, n6 = 2,346 bp/47.3% GC.

**Table 4:** Identified sequences from NGS analysis.

Assembled Sequence	Sequence
1	TAATACGACTCACTATAGGGACAGTCTAGTGCAGCAGTCAGTACGAGTCTCATGAGTGTAGGATGCATGAGATCAACGCTAGCATCGCACTGTCGTCATGCAGCTGACTCCGATCTGACTATCGTCTGAGATCAGAGCGTAACGTAGTCAGTGCTAGCATGCGAACTCGATGATCGAGTCGTATCCACTGTTGCCATATATGCAGACGGCATAGTATGCGTGTATGCGTCGAGAGATCATCCCTATCTTGACGTTAGTTACAAGATCCCACCAATACTGCCAATAGACGGTCCTCCTTTCCCGTTGCTGTAAAACAGTCATGATCGTCATCAGATCATGCCGGCGTGATCTAGATACACGGTGGATTCAGCTACTAGTCGAATCATGACGTGAGAAGCATGAACGATATGAAGAAGTTATGTGGATAGCTGTCGACGTGATCGTATCGATGCAGTCCTCAGGTCATATTACTCGACAGTTGCTAAGTCAGTCATCGTCATACGATGCCGCTGAGCAATAACTAGC
2	TAATACGACTCACTATAGGGACAGTCTAGTGCAGCAGTCAGTACGAGTCTCATGAGTGTAGGATGCATGATCATGATTCTGATCTAGTCCAGCAGTAGAGTCGTCTCGATCGATCTGTGCATCGTCAGCGATATTCGACGTAGTCGCTCGACCTGACTCGTGAGTGCAGCTACGTGTCAGTCATCCACTGTTGCCATATATGCAGACGGCATAGTATGCGTGTATGCGTCGAGAGATCATCCAGTTCTTGACGTTAGTTACAAGATTGGCCACGATCCATGCTAACGTCTCTTCCACCTTTCCCAAAAAGTAACACACCATGACGTATCGACTACGCACATACAGCATATGTGGATGATCACTGACTGACTGAACTACGATCATGGTGTATGTGAGCGTGTATGTGCTCGTGACTGGAGAAACGGCAACAGTGGATGATTGACGTACGACTGCTAGCTCAGGTCATATTACTCGACAGTTGCTAAGTCAGTCATCGTCATACGATGCCGCTGAGCAATAACTAGC
3	TAATACGACTCACTATAGGGACAGTCTAGTGCAGCAGTCAGTACGAGTCTCATGAGTGTAGGATGCATGATCATGATTCTGATCTAGTCCAGCAGTAGAGTCGTCTCGATCGATCTGTGCATCGTCAGCGATATTCGACGTAGTCGCTCGACCTGACTCGTGAGTGCAGCTACGTGTCAGTCATCCACTGTTGCCATATATGCAGACGGCATAGTATGCGTGTATGCGTCGAGAGATCATCCAGTTCTTGACGTTAGTTACAAGATTGGCCACGATCCATGCTAACGTCTCTTCCACCTTTCCCAAAAAGTAACACCGACTGATCGCGCATACGGCAACAGTGACTCTCGACTACCATAGTAGTGAGATGGTGGATTACGATCGCGTGATCTGAGTATCATTGATCTATAGTGGATTGACTGATGATCGTACTGTCGTACTGACTCTGACGTCGATCTCAGGTCATATTACTCGACAGTTGCTAAGTCAGTCATCGTCATACGATGCCGCTGAGCAATAACTAGC
4	TAATACGACTCACTATAGGGACAGTCTAGTGCAGCAGTCAGTACGAGTCTCATGAGTGTAGGATGCATGATCATGATTCTGATCTAGTCCAGCAGTAGAGTCGTCTCGATCGATCTGTGCATCGTCAGCGATATTCGACGTAGTCGCTCGACCTGACTCGTGAGTGCAGCTACGTGTCAGTCATCCACTGTTGCCATATATGCAGACGGCATAGTATGCGTGTATGCGTCGAGAGATCATCCAGTTCTTGACGTTAGTTACAAGATTGGCCACGATCCATGCTAACGTCTCTTCCACCTTTCCCAAAAAGTAACACTGACTGCATTCGTGATCATCATGCCGGCGTGATCTAGATACACGGTGGATTCAGCTACTAGTCGAATCATGACGTGAGAAGCATGAACGATATGAAGAAGTTATGTGGATAGCTGTCGACGTGATCGTATCGATGCAGTCCTCAGGTCATATTACTCGACAGTTGCTAAGTCAGTCATCGTCATACGATGCCGCTGAGCAATAACTAGC
5	TAATACGACTCACTATAGGGACAGTCTAGTGCAGCAGTCAGTACGAGTCTCATGAGTGTAGGATGCATGAGATCAACGCTAGCATCGCACTGTCGTCATGCAGCTGACTCCGATCTGACTATCGTCTGAGATCAGAGCGTAACGTAGTCAGTGCTAGCATGCGAACTCGATGATCGAGTCGTATCCACTGTTGCCATATATGCAGACGGCATAGTATGCGTGTATGCGTCGAGAGATCATCCCTATCTTGACGTTAGTTACAAGATCCCACCAATACTGCCAATAGACGGTCCTCCTTTCCCGTTGCTGTAAAACATAGTCATGACATCGACTACGCACATACAGCATATGTGGATCTAGCTTGACTAGTCAACGTCGATATCGCGTGATCTGAGTATCATTGATCTATAGTGGATTGACTGATGATCGTACTGTCGTACTGACTCTGACGTCGATCTCAGGTCATATTACTCGACAGTTGCTAAGTCAGTCATCGTCATACGATGCCGCTGAGCAATAACTAGC
6	TAATACGACTCACTATAGGGACAGTCTAGTGCAGCAGTCAGTACGAGTCTCATGAGTGTAGGATGCATGAGATCAACGCTAGCATCGCACTGTCGTCATGCAGCTGACTCCGATCTGACTATCGTCTGAGATCAGAGCGTAACGTAGTCAGTGCTAGCATGCGAACTCGATGATCGAGTCGTATCCACTGTTGCCATATATGCAGACGGCATAGTATGCGTGTATGCGTCGAGAGATCATCCCTATCTTGACGTTAGTTACAAGATCCCACCAATACTGCCAATAGACGGTCCTCCTTTCCCGTTGCTGTAAAACATAGTCATGACATCGACTACGCACATACAGCATATGTGGATCTAGCTTGACTAGTCAACGTCGATATCGCGTGATCTGAGTATCATTGATCTATAGTGGATCATGACGTGCATGCAAGCTTAGCTAGTCAGATCAGTAGCTCTCAGGTCATATTACTCGACAGTTGCTAAGTCAGTCATCGTCATACGATGCCGCTGAGCAATAACTAGC

## Conclusions

Rapid advances in DNA synthesis and sequencing technologies are enabling the use of DNA for non-biological applications. One promising application that has emerged is the use of synthetic DNA for data storage, both for communication and long-term data archiving [1]. However, in these early days within the field we advocate exploring different methods—of putting information into, transporting information as, and taking information out of DNA molecules—with the long range goal of attaining a standard that can be accepted by industry and implemented for future applications.

Here we developed a method of encoding information in DNA that reduces the formation of homopolymers by taking into account the frequency of usage of different characters in English text. Our iKey-64 method is designed to convert both plaintext and numerals into a DNA language, while allowing for personalization. Users can shuffle codons assigned to the keyboard or alter the characters within the keyboard to develop a unique layout. With chromatogram patterning and homopolymer reduction, codon shuffling will enable 9.1 x 10^61^ iKey-64 variants out of a maximum of 64! = 1.3 x 10^89^ iKey-64 variants (Fig 3). Furthermore, we developed a secret-sharing system in MuSE that explores fragmentation of messages across multiple distinct DNA molecules, and enables a new method—chromatogram patterning—to locate messages within DNA molecules. Our encoding, transfer, and data extraction methods are proof-of-concept experiments that are designed to explore new approaches of communicating via DNA, and to initiate thought and discussion on how to structure DNA communication channels.

Some limitations of our approach are that the iKey-64 method of encoding has not been tested for encoding numerals, and other methods [7–9] may prove more efficient. The iKey-64 method does not incorporate data compression and it would be interesting to explore ways to adapt data compression methods for encoding using this method. Also, the iKey-64 character-codon assignment was based on the frequenting of usage of characters in the Oxford English Dictionary and with numerals categorized as high frequency characters. While this method was tested for English-based communications, in theory character assignment can be modified for other languages using a similar approach. Additionally, encoding with iKey-64 needs to be combined with other cryptography such as AES, RSA, Twofish and other methods to ensure data security. This will in turn require customized versions of iKey to be designed to allow for the encoding of encrypted data. Moreover, our chromatogram patterning method has been designed for Sanger sequencing. Since we were exploring the fragmented communication of short messages that could be read with a single Sanger read, we chose to use Sanger sequencing for initial proof-of-concept experiments. However, future experiments will need to focus on using NGS sequencing methods as they are more efficient, cost effective, and allow for the investigation of more complex fragmented communications. Additionally, the concept of storing information within overlapping sequencing reads in multiplexed sequencing reactions may also be adaptable for nanopore sequencing and NGS methods.

Thus far our experiments exclusively utilized DNA maintained *in vitro* in the form of plasmid DNA. Maintaining a full set of MuSE plasmids inside the same cell is problematic due to likely segregation loss, as the plasmids share common replication origins and resistance markers [20]. However, DNA maintained *in vivo* can also be used for the communication of digital information, for example for genome watermarking applications [1]. In future experiments, we aim to utilize programmable post-translational protein assembly [21–24] to develop an addiction module that will allow for the intracellular dual maintenance of two plasmids with a common origin and selection marker, which would enable *in vivo* MuSE. We intend for these early explorations to stimulate the development of future DNA communication tools that in turn may further broaden adoption of DNA for communication, an increasing possibility with the development of portable sequencing devices [25,26].
